# Prevalence estimates of diabetes in pregnancy in a rural, sub-Saharan population

**DOI:** 10.1016/j.diabres.2020.108455

**Published:** 2020-11

**Authors:** Alice A. Maidwell-Smith, Andrew M. Doel, Robin M. Bernstein, Sophie E. Moore

**Affiliations:** a Department of Women and Children’s Health, King’s College London, London, UK; b Department of Anthropology, University of Colorado, Boulder, CO, USA; c Health and Society Program, Institute of Behavioural Science, University of Colorado, Boulder, CO, USA; d MRC Unit The Gambia at the London School of Hygiene and Tropical Medicine, Gambia

**Keywords:** Hyperglycaemia in pregnancy, The Gambia, Gestational diabetes, DIP, Diabetes in Pregnancy, GDM, Gestational Diabetes Mellitus, HDIP, Hyperglycaemia first Detected in Pregnancy, HERO-G, Hormonal and Epigenetic Regulators of Growth Trial, HIP, Hyperglycaemia in Pregnancy, IADPSG, International Association of Diabetes in Pregnancy Study Group, IDF, International Diabetes Federation, MRC, Medical Research Council, OGTT, Oral Glucose Tolerance Test, SPSS, Statistical Package for the Social Sciences

## Abstract

**Aims:**

To determine the prevalence of Hyperglycaemia first Detected in Pregnancy (HDIP) in a cohort of women from rural Gambia and compare the diagnostic ability of capillary blood glucose (CBG) sampling to identify HIP versus laboratory-based analysis of venous plasma glucose (VPG).

**Methods:**

Pregnant women from rural Gambia (*N* = 251) underwent a 75 g Oral Glucose Tolerance Test (OGTT) at 28-weeks of gestation. Gestational Diabetes Mellitus was assessed as fasting glucose concentration ≥ 5.1–6.9 mmol/L; ≥10.0 mmol/L at 1-h post load; or ≥ 8.5 mmol/L at 2-h post load and Diabetes in Pregnancy as fasting glucose > 7.0 mmol/L.

**Results:**

A total of 199 and 244 women had VPG and CBG measurements respectively, and 198 women had both. 32 women (16.1%) were diagnosed with HDIP using VPG, mostly based on fasting concentrations.

**Conclusions:**

The prevalence of HDIP in rural Gambia was higher than anticipated, emphasising a need for maternal diabetic policy. Based on the current findings, tailored recommendations could include measuring fasting VPG alone when conducting a full OGTT is not feasible. Similarly, CBG may be of value for excluding disease and thereby limiting costly laboratory-based investigations to a select few.

## 1 Introduction

Hyperglycaemia in pregnancy (HIP), encompassing both diabetes predating pregnancy and hyperglycaemia first detected in pregnancy (HDIP), affects 15.8% of live births worldwide [1]. Affected pregnancies have increased risks of adverse perinatal outcomes, including preterm labour, caesarean section and neonatal hypoglycaemia [2], [3], [4], [5]. Furthermore, a previous history of HIP infers risk of adverse long-term maternal and offspring cardiometabolic disease [6], [7].

Data on HIP prevalence estimates in sub-Saharan Africa is limited [8], [9]. The International Diabetes Federation (IDF) estimate HIP occurs in 9.6% of women in Africa, affecting 3.5 million live births annually [1]. However, and as is the case for many of the global estimates, available literature for sub-Saharan Africa uses different screening and diagnostic approaches and is, therefore, insufficient to establish the true prevalence [3], [8], [9], [10].

Recent prevalence estimates using the International Association of Diabetes in Pregnancy Study Group (IADPSG) diagnostic criteria observed a prevalence of HDIP of 8.6% in Nigeria, and 13.1% in Tanzania [11], [9], [12]. However, there remains a lack of data from sub-Saharan Africa, particularly in rural areas, which must be addressed due to the emerging epidemic of metabolic disease in the region [1], [13], [14].

The IADPSG recommends that all women, irrespective of risk factors, should undergo a single step 2-h 75 g Oral Glucose Tolerance Test (OGTT) using venous plasma glucose concentrations at 24–28 weeks of gestation to identify hyperglycaemia [11]. This offers a practical laboratory-based means of diagnosing HIP within high income countries. However, little is known about the applicability of these criteria to women in sub-Saharan Africa who are underrepresented in the evidence base. Furthermore, translation to resource restricted settings may not be feasible because access to suitable facilities for obtaining blood samples, and their subsequent transport, storage and testing, may be limited. This is especially relevant if international guidelines on the laboratory measurement of venous plasma glucose are to be achieved [15]. In light of this, capillary blood glucose sampling using a plasma-calibrated portable glucometer has been proposed as a practical and affordable alternative to venous plasma glucose within resource restricted settings [3].

In comparison to venous blood sampling and plasma glucose measurements, use of capillary samples and glucometers is minimally invasive and requires comparatively little training to use. However, glucometers are less technically accurate when compared to references values of venous plasma glucose [16]. Recent comparisons of the diagnostic ability of capillary sampling versus gold-standard venous sampling reported a sensitivity and specificity of 62.3% and 80.7% in southern India [17], 57% and 91% in western Kenya [18], and 27% and 89% in South Africa [19]. Notably, literature may be pervasively limited by regarding venous plasma glucose as the reference standard, which could itself lack diagnostic accuracy if sample preparation and processing is not optimised [20], [21]. Indeed, the sensitivity and specificity of capillary sampling emerged superior to non-optimised venous sampling when both techniques where compared to optimised venous plasma glucose measurements (51% and 100%, vs. 37% and 100%) [22].

The objectives of the current study were, firstly, to determine the prevalence of HDIP, including Gestational Diabetes Mellitus (GDM) and Diabetes in Pregnancy (DIP), within a cohort of women in rural Gambia according to the current IADPSG diagnostic criteria and, secondly, to compare the diagnostic ability of capillary blood glucose sampling to identify HDIP versus gold-standard laboratory-based analysis of venous plasma glucose.

## 2 Methods

This study uses observational data collected as part of a longitudinal cohort study looking at the Hormonal and Epigenetic Regulators of Growth (HERO-G). The primary focus of HERO-G is infant growth from birth to two years of age; the analysis presented here is limited to data collected during pregnancy, and presents a secondary analysis of data collected as part of HERO-G. The full HERO-G protocol is described elsewhere [23].

All women of reproductive age (18–45 years) living in the West Kiang region of The Gambia were identified using the West Kiang Demographic Surveillance System [24]. Women suitable for inclusion were invited to participate in the study between February 2014 and March 2015. Exclusion criteria were specified as: (1) HIV antibody positive or refusal of HIV testing; (2) major congenital malformations; (3) chronic diseases including sickle cell disease and asthma; (4) already known pregnancy > 28 weeks of gestation at recruitment; and (5) multiple pregnancy.

Consenting women were visited monthly, and asked about the date of last menstrual period. If there was no clear reason for missed menses, women were asked to provide a urine sample for pregnancy testing. If the test indicated a pregnancy, women were then invited to an antenatal booking visit the following week at the Medical Research Council (MRC) field station in the main village of Keneba.

At booking, pregnancy status was confirmed by ultrasound, gestational age ascertained, a routine antenatal examination conducted, and maternal anthropometry (height and weight) collected. Previous maternal medical and obstetric histories were also recorded, where known.

At 28 weeks of gestation, all women were invited to undergo a 75 g OGTT following an overnight fast of at least 12-h duration. Measurements of blood glucose level were recorded at baseline (fasting plasma glucose), which was immediately prior to administration of the oral glucose load (75 g *Dexola* or *Glucola* solution), and at 1-h and 2-h following glucose consumption. Capillary blood glucose levels were first measured using finger prick samples tested with an Accu-Chek® glucometer (Roche Diabetes Care, Hertfordshire, UK). Glucometers were regularly calibrated against low and normal control samples, and all tests were performed within the laboratory environment at room temperature, by trained operatives, and following manufacturers’ guidelines. Samples of venous blood were also collected by venepuncture into Lithium Heparin prepared Monovettes (Sarstedt Ltd, Leicester, UK), stored on ice until centrifugation (3000RPM for 10 minutes at 4 °C) within an hour of collection. Plasma was stored in aliquots for subsequent laboratory analysis of plasma glucose using a Cobas® Analyzer (Cobas Integra 400 plus, Roche Diagnostics, West Sussex, UK). Where results indicated HIP, women were referred to the study midwife for follow up. Typically, this included repeat clinic visits where blood glucose levels were monitored, alongside guidance on dietary approaches to manage blood glucose levels.

The primary outcome assessed was the prevalence of HDIP, including both GDM and DIP, according to the IADPSG diagnostic criteria. The IADPSG specify GDM as at least one of: fasting glucose concentration ≥ 5.1–6.9 mmol/L; ≥10.0 mmol/L at 1-h post load; and ≥ 8.5 mmol/L at 2-h post load. DIP is specified as fasting glucose concentration > 7.0 mmol/L at [11].

The diagnostic accuracy of capillary blood glucose versus venous plasma glucose was considered as a secondary outcome. As a secondary outcome, the study was not specifically designed with this comparison in mind. As a consequence, processing of the venous plasma samples did not meet the strict, international guidelines for the VPG samples to be considered as a true gold standard, such as ensuring all samples were processed within a 30 minute time window [15], [25]. However, this comparison was still considered of value within the current health setting and was assessed using paired sample T-test, constructing Bland-Altmann plots, and Chi-squared test of independence. Finally, the sensitivity and specificity of capillary sampling was determined using 2 by 2 matrices.

Statistical analyses were performed using Microsoft Excel 2017 (version 16.11.1) and the Statistical Package for the Social Sciences (SPSS) software (version 24.0).

Ethical approval for the study was given by the joint Gambia Government/MRC Unit The Gambia Ethics Committee (SCC 1313v3), with additional approval from the University of Colorado Institutional Research Board (protocol number 13–0441). Prior to the start of the study, community approval was obtained from each participating village, and written, informed consent obtained from each participant.

## 3 Results

A total of 1,669 women from across the West Kiang region were eligible for inclusion in the HERO-G study; of these 1,392 consented to participate [23]. Baseline socio-demographic characteristics for those enrolled are presented in Table 1. There were no previous, clinically confirmed cases of GDM among the women recruited.

**Table 1:** Socio-demographic characteristics of study participants.

		Value a	Range
**Maternal age at delivery (years)**		30.4 (6.8)	18–44
**Weight (kg)**		59.5 (10.8)	41.2–99.1
**Height (cm)**		162.4 (5.3)	147.4–176.5
**BMI (kg/m^2^)**		22.5 (3.8)	15.5–36.9
**Systolic blood pressure (mmHg)**		107.8 (9.3)	85.0–135.3
**Diastolic blood pressure (mmHg)**		65.1 (8.4)	43.0–89.7
**Duration of English schooling (years)**		1.3 (3.2)	0–12
**Duration of Arabic schooling (years)**		1.1 (2.9)	0–12
**Number of previous pregnancies**	0	11.4%	0–13
	1–5	50.3%	
	6–10	36.3%	
	>10	2.0%	
**Number of children alive**	0	12.6%	0–12
	1–5	61.1%	
	6–10	25.7%	
	>10	0.6%	

a Values are given as mean (±SD) for continuous variables, or percentage for categorical variables.

**Fig. 1:**
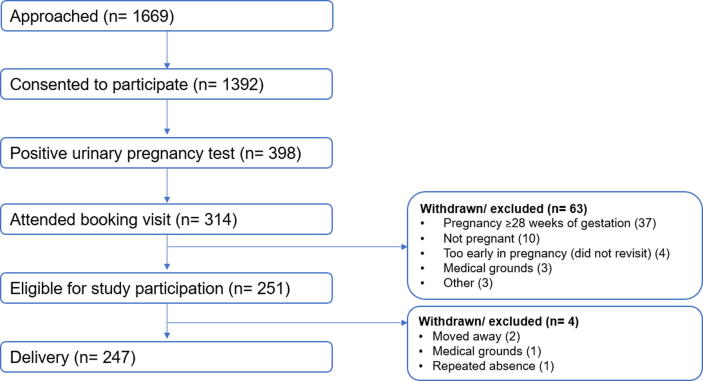
CONSORT diagram showing flow of participants with reasons for withdrawal/ exclusion.

Of the 314 women entered into the pregnancy phase of HERO-G, 251 were eligible for participation in this study. 199 and 244 women had venous and capillary glucose concentrations recorded at their 28-week antenatal visit, respectively. 198 women had both venous and capillary measurements available. There was a delivery recorded for 247 women. The flow of study participants with reasons for exclusion or withdrawal is shown in Fig. 1.

32 women were identified with HDIP by venous sampling, including both GDM and DIP, giving an estimated prevalence of 16.1% (95% CI 11.0–21.2). Most women were diagnosed using fasting blood glucose thresholds, as shown in Table 2. On further classification, 29 women (14.6%, 95% CI 9.7–19.5) had GDM, whilst a further 3 women (1.5%, 95% CI −0.2–3.2) had sufficient glucose intolerance to warrant diagnosis of DIP.

**Table 2:** Prevalence of GDM and DIP according to venous and capillary blood sampling.

	Prevalence of GDM*		Prevalence of DIP**	
	*Venous*	*Capillary*	*Venous*	*Capillary*
**At all time intervals**	14.6% (n = 29)	13.9% (n = 34)	1.5% (n = 3)	1.6% (n = 4)
**Baseline (fasting)**	11.7% (n = 23)	7.9% (n = 19)	1.5% (n = 3)	1.7% (n = 4)
**1 h**	4.0% (n = 8)	5.3% (n = 13)	–	–
**2 h**	4.1% (n = 8)	6.2% (n = 15)	–	–

* IADPSG criteria specify GDM as 1 + of: glucose concentration ≥ 5.1–6.9 mmol/L at baseline (fasting); ≥10.0 mmol/L at 1 h post-load; and ≥ 8.5 mmol/L at 2 h post-load.

** IADPSG criteria specify DIP as glucose concentration > 7.0 mmol/L at baseline (fasting).

38 women were identified with HDIP using capillary sampling, producing a total prevalence of 15.5% (95% CI 11.0–20.1). Of these, 34 women were diagnosed with GDM (13.9%, 95% CI 9.6–18.2) and 4 women diagnosed with DIP (1.6%, 95% CI 0.0–3.2). The greatest number of women were diagnosed on the basis of their fasting capillary blood glucose level; Table 3. Presence of HDIP was not associated with maternal age, parity or BMI at enrolment into the study (p > 0.05 for all; analyses not presented).

**Table 3:** Comparison of women diagnosed with HIP by venous and capillary blood sampling, where both measurements were available.

		Venous plasma glucose (Reference standard)		
		*Diagnosed*	*Not-diagnosed*	*Total*
Capillary blood glucose	*Diagnosed*	22	8	30
	*Not-diagnosed*	10	158	168
	*Total*	32	166	198

**Fig. 2:**
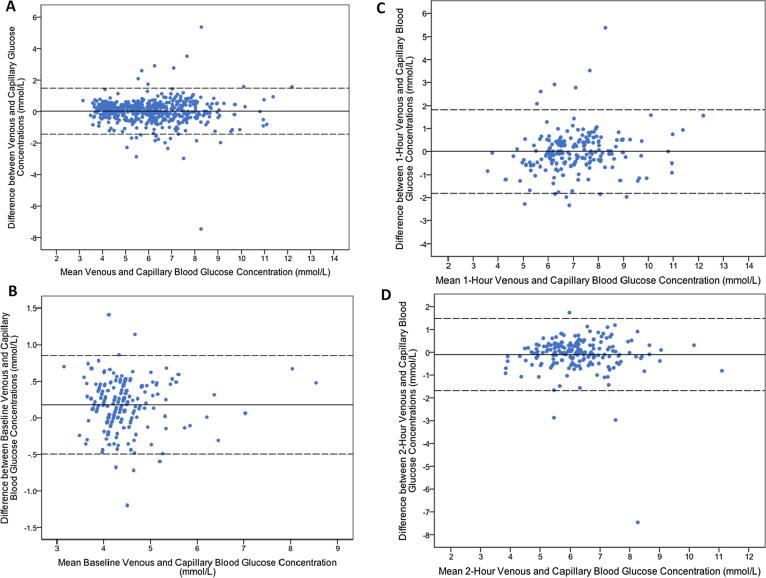
Bland-Altman plots showing the difference between venous and capillary blood glucose concentrations at (A) all time intervals, (B) baseline (fasting), and (C) 1-h and (D) 2-h after 75 g glucose load.

Comparison of the venous and capillary blood glucose concentrations using Bland-Altman plots indicated a mean difference of 0.03 mmol/L (95% CI −1.43–1.49) at all time intervals, 0.18 mmol/L (95% CI −0.50–0.85) for fasting levels, 0.01 mmol/L (95% CI −1.82–1.83) at 1-hour post load, and −0.10 mmol/L (95% CI −1.68–1.48) at 2-h post load (Fig. 2).

Mean fasting venous plasma glucose concentration was significantly different from fasting capillary blood glucose concentration (4.52 vs. 4.33 mmol/L; 95% confidence internal (CI) 0.13, 0.23, p < 0.05). However, there was no significant difference between venous and capillary sampling at either 1-hour (7.11 vs. 7.10 mmol/L; 95% CI −0.13, 0.14, p = 0.94) or 2-hours (6.23 vs. 6.33 mmol/L; 95% CI −0.22, 0.01, p = 0.09) post load.

22 women were diagnosed with HDIP by both venous and capillary blood glucose measurements, with the remaining women either being diagnosed by venous but not capillary blood sampling (n = 10), or capillary but not venous blood sampling (n = 16). The proportion of women diagnosed with venous sampling differed significantly from the proportion diagnosed using capillary sampling (χ2 (1) = 80.39, p < 0.001). Using venous sampling as the reference standard, capillary sampling had a sensitivity of 68.8% and specificity of 95.2% (Table 3).

## 4 Discussion

In this population of rural African women, we estimated a prevalence rate for hyperglycaemia first detected in pregnancy of 16.1% when using gold-standard laboratory analysis of venous plasma glucose. Adoption of IADPSG diagnostic thresholds for further classification, revealed a total prevalence of 14.6% and 1.5% for GDM and DIP respectively. These are the first published estimates of GDM and DIP rates for The Gambia. Furthermore, this research supports the use of fasting plasma glucose levels alone, or the use of capillary blood glucose; both presenting feasible options in resource-limited settings where conducting a full oral glucose tolerance test or collecting and processing venous blood samples may not be possible.

In 2019, the International Diabetes Federation estimated the prevalence of any form of HIP to be 15.8% globally and 9.6% in Africa [1]. Notably, the burden of metabolic disease is forecasted to rise at an alarming rate – the number of people with diabetes in general is predicted to rise by 142.8% by 2045 in Africa [1]. Despite this, literature pertaining to metabolic disorders of pregnancy in sub-Saharan Africa is lacking. Recent literature using IADPSG criteria to determine the prevalence of HDIP has, however, reported a lower prevalence of 8.6% in Nigeria, and 13.1% in Tanzania [12], [26]. These data from rural Gambia provide an important contribution to the existing literature.

The reported prevalence was similar to IDF estimations of the global prevalence of any form of HIP (15.8%) [1]. However, the observed prevalence of HDIP exceeded estimations from other recent literature using IADPSG criteria in sub-Saharan Africa [12], [26]. Notably, previous studies have predominantly focused on urban and semi-urban African populations [12], [26], which are at greater risk of glucose intolerance than rural populations [14], [27]. In fact, the prevalence of HDIP in urban and rural Tanzania varied by 14% (17.7% vs. 3.7%) [26]. With this is mind, it is surprising that the prevalence of HDIP reported by the current work, detailing findings from a rural population, is comparable to previous estimates of urban populations. In the current cohort studied 12.9% (n = 32) of women were overweight (25.0–29.9 kg/m^2^) and an additional 5.6% (n = 14) were obese (≥30 kg/m^2^), which is greater than previous estimates for this population but still below many estimates for urban African populations [28]. Similarly, the mean maternal age in the current cohort (30.4 years) was older than in other literature estimating the prevalence in rural areas (26.7 years) [26], and a greater proportion of women were ≥ 35 years of age in this study (32.8%) than in previous literature (18.8%) [12]. Mean parity was also relatively high at 4.4. However, within this cohort, neither maternal BMI, age or parity at entry into the study were related to risk of HIP suggesting that these risk factors were not driving HDIP risk within this context.

Previous research has excluded women with known diabetes [12], [26]. In this study, women with any diagnosed chronic condition (including diabetes) were similarly excluded at the point of participant recruitment. However, because screening for diabetes was not done at recruitment, it is possible that some women may have entered the study with undiagnosed diabetes or been diagnosed after enrolment. As a result, the reported prevalence of HDIP in the current work may in fact represent any form of HIP, including pregnancy-related (gestational) and pre-existing diabetes. This may be an additional reason why the apparent prevalence of HDIP in this study exceeds previous estimates. However, the proportion of women with overt diabetes misclassified as HDIP is likely to be small as the prevalence of DIP, which is based on the same glucose thresholds for overt diabetes in non-pregnant adults, was low (1.5%). Therefore, the overall implications of not excluding those with pre-existing diabetes may be minimal.

Of relevance, most women (79.3%) were diagnosed on the basis of their fasting venous plasma glucose concentrations. This is important because using a full 2-hour 75 g OGTT for diagnosis may not always be achievable in low resource settings. Instead, there may be value from using fasting venous glucose concentrations alone to identify hyperglycaemia in pregnant women. This observation is strongly consistent across the evidence base employing IADPSG criteria [12], [19], [21]. However, it is important to recognise that 20.7% of women in this study would not have been diagnosed with HDIP had fasting venous sampling been used in place of an OGTT. Therefore, considering the adverse outcomes associated with unmanaged HIP, it is uncertain whether the clinical and cost effectiveness of fasting venous sampling would be superior to conducting a full OGTT.

This study demonstrated blood glucose concentrations measured using capillary and venous sampling were comparable. However, there were discrepancies in the women identified using the different methods. This may have arisen from accepting conventional laboratory analysis of venous plasma glucose as the reference standard when evaluating the diagnostic ability of capillary blood sampling. Capillary sampling may be superior to laboratory-based venous sampling if time to centrifugation is not optimised, defined as > 30 minutes [22]. Indeed, in this study venous samples were managed within an hour of collection, which does not fulfil ideal collecting conditions [22]. Therefore, comparing the diagnostic ability of capillary and non-optimised venous sampling may make capillary sampling falsely appear to perform poorly. As such, capillary sampling should not be considered less clinically accurate for diagnosing HDIP on the basis of this study alone.

In contrast, this study revealed capillary sampling identified those without HDIP accurately, which is in accordance with previously reported high diagnostic specificity (95.2% vs. 80.7–100%) [21], [17], [18], [19]. Therefore, capillary sampling may be clinically useful to exclude disease. This observation is of relevance for low resource settings as it implies capillary sampling could be used to exclude those unlikely to have hyperglycaemia from undergoing costly laboratory-based investigations. This offers a significant contribution to the field and warrants future investigation and cost-benefit analysis before being appropriate to recommend for clinical practice.

The current work is of importance because it adds to the existing limited data from this population and setting. A key strength of this study is a detailed description of the study population, which will be valuable for comparison with further literature. Furthermore, controlled administration of the OGTT and standardised blood sampling within a single clinic allowed external factors that may reduce the accuracy of measurements, and introduce detection bias, to be minimised. Finally, the availability of both venous and capillary sampling provided a unique opportunity to compare measurements.

The current work also has several limitations. Firstly, small sample size, further compounded by the infrequent occurrence of HDIP, meant statistical evaluations may lack power. Secondly, women with undiagnosed but pre-existing overt diabetes were not excluded, which may result in misclassification of overt diabetes as HDIP and subsequently overestimate the prevalence. Thirdly, while all efforts were made to ensure fasting at time of sampling (women were collected from their homes early in the morning, and before the first meal of the day – which is typically mid-morning), the community nature of the study cannot guarantee this. Fourthly, the lack of adherence to the strict protocols required for collection and processing of venous blood samples for glucose analysis (specifically, extending the time window from collection to processing from the recommended maximum of 30 minutes to less than an hour) limits our ability to claim to venous blood samples as a true gold standard [15], [25]. This impacts both prevalence estimates and the validity of direct comparison of venous with capillary blood glucose samples. Further, these data are only reflective of the women recruited into the HERO-G study and may not be representative on a population or even regional basis (e.g. women who presented > 28 weeks’ gestation were excluded from the HERO-G study). Finally, whilst this study improves understanding of HDIP in sub-Saharan Africa, the extent to which findings can be validly generalised to other settings is limited as they are exclusively representative of rural Gambia.

## 5 Conclusions

The prevalence of HDIP (16.1%) within this population of women in rural Gambia was higher than anticipated. Measuring fasting venous plasma glucose sampling alone may be of diagnostic potential where conducting a full OGTT is not feasible, such as in a resource-limited setting. There was strong agreement between capillary blood glucose and venous plasma glucose concentrations, although the diagnostic accuracy of capillary sampling versus venous sampling could not be established because the laboratory conditions for handling venous samples were not optimised. Importantly, capillary sampling may be of value for excluding disease and limiting costly laboratory-based investigations to a select few.

## Funding

The HERO-G study was funded by the Bill and Melinda Gates Foundation (OPP1066932).

## Declaration of Competing Interest

The authors have no competing interests to declare.
